# Supplementary material for: Selective reinforcement of conflict processing in the Stroop task
Source: PLoS One. 2021 Jul 30;16(7):e0255430. doi: 10.1371/journal.pone.0255430 (PMC8323904; doi:10.1371/journal.pone.0255430)
Supplement: S1 Appendix — (DOCX) [file pone.0255430.s001.docx]

**S1 Appendix. Instructions presented to the participants during the experiments.** After an introductory message, participants received the following instructions: “During the experiment, words that represent names of colors will be shown on the screen (e.g., BLUE). In some trials, the ink-color and the word will be congruent, while in others trials the ink-color and the word will be incongruent. Each time a word is shown on the screen, your task will be to indicate the ink-color of the word. The ink-color can be red, green, blue, or yellow. To indicate the ink-color of the word shown on the screen, we will ask you to press one of the four keys of the keyboard: S, D, J, or K, while the word is shown. Each key S, D, J, or K, is matched with one of the four ink-colors. During the experiment, each new trial will be signaled by a cue shown on the screen (a triangle or a circle). We will ask you to not respond when the cue is shown, but to respond only when a word stimulus is shown.” And before practice trials, participants received the following instructions (here for practice on congruent trials): “Next, you will start a training on congruent trials, during which the ink-color and the word shown are similar. A grey square shown on the screen just after you responded indicates that your response was correct. The absence of the grey square after you responded indicates that your response was incorrect.” Finally, participants received the following instruction for additional monetary reward: “Sometimes the symbols $ and # will be shown on the screen. Each time the symbol $ is shown, it means that you earned 2 cents. Your reward bonus after the experiment will depend on how many times you saw the symbol $.”
